# Plant-Based Cannabinoids for the Treatment of Chronic Neuropathic Pain

**DOI:** 10.3390/medicines5030067

**Published:** 2018-07-01

**Authors:** Sherelle L. Casey, Christopher W. Vaughan

**Affiliations:** Pain Management Research Institute, Kolling Institute of Medical Research, Northern Clinical School, Royal North Shore Hospital, University of Sydney, Sydney, New South Wales 2065, Australia; chris.vaughan@sydney.edu.au

**Keywords:** cannabinoids, *Cannabis sativa*, phytocannabinoids, neuropathic pain, delta-9-tetrahydrocannabinol (THC), cannabidiol

## Abstract

Chronic neuropathic pain is a prevalent condition that places a heavy burden on individuals and the healthcare system. Current medications have limitations and new approaches are needed, particularly given the current opioid crisis. There is some clinical evidence that the plant *Cannabis sativa* produces relief from neuropathic pain. However, current meta-analyses suggest that this efficacy is limited and there are problems with side effects. Most of this clinical research has examined whole cannabis, the psychoactive phytocannabinoid 9-tetrahydrocannabinol (THC), and nabiximols, which are a mixture of THC and the non-psychoactive phytocannabinoid cannabidiol. In the past, there has been little evidence based, preclinical animal research to guide clinical studies on phytocannabinoids. Recent animal studies indicate that while THC and high dose nabiximols are effective in animal neuropathic pain models, significant pain relief is only achieved at doses that produce substantial side effects. By contrast, cannabidiol and low dose nabiximols have moderate pain relieving efficacy, but are devoid of cannabinoid-like side effects. This animal data suggests that cannabidiol and low dose nabiximols warrant consideration for clinical studies, at least as adjuvants to current drugs. Preclinical research is also required to identify other phytocannabinoids that have therapeutic potential.

## 1. Introduction

Chronic pain, or pain persisting for longer than the time expected for tissue healing, is a prevalent and costly condition that places a large burden on the healthcare system. Chronic pain affects approximately 40% of adults in the United States of America, with an estimated annual cost of up to $635 billion [1]. Neuropathic pain is a particularly debilitating form of chronic pain arising from damage to the central or peripheral nervous systems, which can be caused by physical trauma (for example, accidents, surgery, and stroke), diseases including diabetes, cancer, and immune disorders, and medications such as cancer chemotherapy agents [2]. It is a severe abnormal pain syndrome, generally characterized by spontaneous pain (pain in the absence of any stimulus). Sufferers may also experience mechanical and/or thermal allodynia, abnormal pain states whereby normally innocuous stimuli such as light touch or stroking (for example from putting on clothing), or mild temperate changes (such as showering) are perceived as painful. Hyperalgesia, or an increased pain response to a usually mildly painful stimulus, may also occur [3,4]. In addition to pain, many neuropathic pain patients also suffer from comorbid psychosocial disturbances including depression, anxiety, sleep disorders, and reduced social interactions [5].

Compounding these problems, neuropathic pain is extremely difficult to manage, with currently available pharmacotherapies providing limited pain relief and often having disabling side effects including dizziness, sedation, depression, and sleep disorders, rendering them intolerable to many patients [6,7]. Indeed, at least 50% of neuropathic pain sufferers do not obtain clinically meaningful pain relief from current therapeutic options [7]. These limitations highlight the need for new therapeutic options, either as first-line medications or as adjuvants to enhance current therapies. This narrative review summarizes the literature regarding the use of cannabinoids for the treatment of neuropathic pain.

## 2. Cannabis

The plant *Cannabis sativa* has been used for millennia for treatment of a variety of conditions including sleep disorders, pain, anxiety, depression, stress, and neuralgia [8,9]. It has recently received a lot of media and political attention as an alternative treatment option for chronic diseases including pain, multiple sclerosis, and epilepsy. The *Cannabis sativa* plant contains over 400 different compounds, including phytocannabinoids and other compounds. However, the majority of clinical and preclinical research has focused on whole cannabis and its two major phytocannabinoid extracts, delta-9-tetrahydrocannabinol (THC) and cannabidiol, either alone or in combination. A number of preclinical studies have shown that phytocannabinoids produce some of their physiological effects by acting on an endogenous neurotransmitter system comprising endogenous cannabinoids (endocannabinoids), a class of G-protein coupled receptors (CB1 and CB2 receptors), and systems for their production and degradation [10]. The role of the endocannabinoid system in these studies has traditionally been assessed using synthetic cannabinoid receptor agonists, selective cannabinoid receptor antagonists, and cannabinoid receptor knockout animals [11].

THC is the primary psychoactive component of *Cannabis sativa* and has beneficial analgesic, anti-inflammatory, and anti-emetic effects; however it also produces side effects including cognitive impairment, dizziness, sedation, motor incoordination, anxiety, dry mouth and/or eyes, and psychosis [12,13]. Cannabidiol is the primary non-psychoactive component of *Cannabis sativa* and has anti-inflammatory, neuroprotective, anxiolytic, and anti-psychotic actions, but does not produce the typical cannabinoid side effects [12,14]. While the actions of THC are at least partly mediated by CB1 and CB2 receptors, the actions of cannabidiol are only partly mediated by CB2 receptors, with other receptor systems having a major role in its activity [10,15].

## 3. The Clinical Evidence for Cannabinoid Efficacy against Neuropathic Pain Is Poor

To date, clinical studies have largely focused on phytocannabinoids, including whole cannabis, THC or its synthetic analogue (dronabinol), or nabiximols which are combinations of THC and cannabidiol (e.g. Sativex) to treat neuropathic pain of various origins. In these studies, the phytocannabinoids are most commonly delivered by inhalation (vaporized, smoked), orally, or oromucosally.

During the first 10 years of the millennium, a number of studies investigated the effect of smoked cannabis products for relief of neuropathic pain, including naturalistic and observational studies as well as randomized controlled trials. The findings of a 2005 observational study suggest that cannabis is useful for the treatment of neuropathic pain and is better tolerated by patients when compared to prescription medication [8]. It has been suggested that cannabis cigarettes relieve HIV-associated neuropathic pain at a comparable level to prescription medications in patients with pain refractory to other treatments [16,17]. These findings are supported by a study evaluating efficacy of smoked cannabis for neuropathic pain, with 3.5% and 7% THC cigarettes providing analgesia superior to placebo [18].

More recently, clinical trials have focused on vaporization, whereby active cannabinoids are released without harmful compounds associated with carbon combustion [16]. Similarly to smoked cannabis, studies examining vaporized cannabis at various doses suggest that it is effective for treatment of neuropathic pain [18,19]. Of note is the finding in many of these studies that low-dose THC is equally as effective as medium and high doses, with reduced frequency and intensity of side effects in the low dose groups, suggesting that it is possible to provide analgesia without adverse side effects.

Interestingly, it has been suggested that co-administration of THC and cannabidiol may enhance therapeutic benefits while attenuating undesirable THC side effects [20,21,22]. Many clinical trials support this notion [12,14,23]. For example, it has been shown that cannabis cigarettes containing higher levels of cannabidiol produce less of the memory impairment and cognitive deficits associated with THC [14]. However, the interactions between THC and cannabidiol are highly variable and appear to depend upon dose ratios and the experimental measure being evaluated [24]. Thus, there is no strong evidence for synergistic, or even additive beneficial effects of these cannabis constituents.

Given that nabiximols (oromucosal sprays containing both THC and cannabidiol) have received regulatory approval in many countries, the safety and convenience of the oromucosal administration route, and the evidence for modulation of adverse THC side effects by cannabidiol, there is a body of clinical research evaluating THC and cannabidiol in combination. Many studies evaluating nabiximols suggest that they provide effective analgesia (reduction in pain intensity and allodynia) in addition to helping with sleep and disability associated with pain. They are usually well-tolerated by patients, with side effects generally being described as mild to moderate and not significantly impacting on day-to-day functioning [25,26,27]. Of note is the fact that tolerance does not seem to develop in open-label extension studies, with benefits being maintained for up to four years on a stable dose [23]. It has also been suggested that more patients may benefit from long term use when compared to short term use [25,28]. However, other studies suggest that nabiximols are not useful for treatment of neuropathic pain, with findings suggesting that despite patients reporting improvement in sleep quality and global impression of change, there is no significant reduction in pain [29]. Another study reported conflicting results in the two phases of the study, and although there were no significant benefits compared to placebo in the treatment phase, nabiximols provided analgesia (>30% pain improvement), improved sleep, and maintenance of analgesia compared to placebo in the withdrawal phase. It is of note that patients who stopped THC/cannabidiol therapy did not experience withdrawal syndrome [30]. Another study reported no significant difference in mean pain score or pain intensity between THC/cannabidiol treatment and placebo, although this finding is questionable as depression was a major confounder [31].

While the above studies suggest that cannabis may be useful for chronic neuropathic pain, it is important to note that the most up-to-date meta-analysis of cannabinoids suggests that they have some, but only modest, efficacy against chronic pain [32,33,34,35,36]. The problem, however, is that such a cannabinoid meta-analysis does not always distinguish between types of cannabinoids used in these studies, or their route, dose, or duration of administration. This might be contrasted to a meta-analysis of a specific drug, such as gabapentin or pregabalin, which only evaluates studies that use specific doses and routes of administration. In addition, such a meta-analysis does not always distinguish between disease types/subtypes and their severity, or whether patients are resistant to current drugs. This information may be crucial in determining the most effective use of cannabinoids.

## 4. How Does This Correlate to Preclinical Animal Studies

Given the paucity of clinical evidence in favor of cannabinoids, it is somewhat surprising that there is a very large body of preclinical animal evidence that synthetic cannabinoid agonists are highly effective for the treatment of neuropathic pain [6]. Unlike clinical trials, however, far fewer preclinical studies have examined the actions of phytocannabinoids. Nonetheless, a small group of studies have demonstrated that THC and cannabidiol, either alone or in combination, have pain relieving efficacy in a range of animal neuropathic pain models.

### 4.1. THC

THC has been shown to abolish the mechanical and thermal allodynia associated with various neuropathic pain models in rodents [15,37,38,39,40]. However, in addition to analgesia, THC also produces classic cannabinoid side effects such as sedation, catalepsy, reduced locomotion, and hypothermia [21,24,26,41]. The question therefore arises, “why has this not translated into clinical studies?”

One important issue with these studies is that they rarely determine the window between the doses at which drugs produce analgesia and side-effects. We have recently shown that THC has a therapeutic index of approximately 5–6, i.e., it produces analgesia with an ED_50_ 5–6 times lower than that at which it produces side-effects [15]. Unfortunately, this is a relatively small separation between analgesia and side-effects, albeit better than synthetic cannabinoid agonists (such as WIN55212) which have therapeutic indices of near unity, i.e., no window between analgesia and side-effects [42,43,44].

### 4.2. Cannabidiol

Like THC, cannabidiol has been shown to reduce the pain associated with various neuropathic pain models in rodents [15,38,40,45,46,47]. However, it should be noted that cannabidiol has a maximal analgesic effect (efficacy) that is only half of that observed for THC [15]. Unlike THC, cannabidiol does not produce cannabinoid-like side effects even at high doses, which suggests that cannabidiol has a very wide therapeutic window [15]. It might also be noted that some studies have shown that the analgesic efficacy of cannabidiol improves with chronic treatment [45]. Thus, cannabidiol presents a potential alternative therapy that has not been explored in clinical studies.

### 4.3. Combination THC and Cannabidiol (Nabiximols)

Given the widespread belief that nabiximols may offer chronic pain relief with fewer side effects, it is surprising that, until last year, no preclinical studies had investigated the interaction between THC and cannabidiol in a neuropathic pain state. It has recently been shown that THC and cannabidiol administered in a 1:1 ratio act synergistically to prevent the development of chemotherapy-drug induced neuropathic pain in mice [40]. In addition, we have recently demonstrated a highly synergistic interaction between THC and cannabidiol in reducing allodynia associated with a nerve injury model in mice [15]. It is of note that this synergy did not extend to the side effects of THC, with the side effect profile of the THC/cannabidiol combination being similar to that for THC alone. The results of these studies indicate that THC/cannabidiol combination treatment has a very large therapeutic window [15].

There are two important caveats arising from the above preclinical study [15]. Firstly, the analgesic efficacy of the THC/cannabidiol combination at low doses is only 30–60% of that of THC. This is similar to that observed for cannabidiol alone. Secondly, the analgesic and side effect profile of the THC/cannabidiol combination at high doses is virtually indistinguishable from that of THC alone. Thus, co-administration of THC and cannabidiol may only offer a superior option to THC at low doses, and even then it might be better as an adjuvant to current therapies.

### 4.4. Comparing the Phytocannabinoids

The above preclinical studies indicate that while the major psychoactive component of cannabis, THC, efficaciously reduces neuropathic pain, it has a relatively small therapeutic window. This will lead to the emergence of significant side effects at any dose that produces meaningful pain relief, and indeed, this is reflected in a number of clinical studies. The major non-psychoactive ingredient of cannabis, cannabidiol, differs from THC in its relative benefits and risks. While cannabidiol has lower efficacy against neuropathic pain, it displays an excellent therapeutic window, at least in terms of cannabinoid-like side effects. To date, there are no major clinical studies on the effectiveness of cannabidiol for the treatment of neuropathic pain. Finally, the benefits of combination THC/cannabidiol therapy only become evident at low doses where it is more likely to provide a low efficacy adjuvant to other therapies. At high doses, the animal evidence suggests that combination THC/cannabidiol treatment will offer no advantages over THC.

## 5. Conclusions

While there is clinical evidence that cannabis and its extracts have some efficacy against chronic neuropathic pain, the current meta-analyses suggest that this efficacy is relatively modest compared to placebo. It might be noted, however, that cannabis and cannabinoids consistently improve sleep and disability index despite the fact that they do not always significantly improve pain. Cannabinoids are generally well-tolerated by patients and there is good evidence to suggest that tolerance does not develop to their beneficial effects. In fact, a higher proportion of patients respond better to longer term treatment, suggesting that cannabinoids may offer a safer alternative than some current treatments.

It is unclear from the meta-analyses of clinical studies which phytocannabinoids or synthetic cannabinoids should be pursued. While such a decision requires evidence based research, preclinical animal studies have only recently systematically examined the effectiveness of phytocannabinoids in neuropathic pain states. These studies indicate that THC and a high dose THC/cannabidiol combination are not ideal options for neuropathic pain. By contrast, a low dose THC/cannabidiol combination and even high dose cannabidiol have potential, and neither of these have been examined in clinical trials. It is also possible that other phytocannabinoids in the cannabis plant might provide therapeutic options, although these would have to be first evaluated in preclinical animal studies.
